# The battered who commit homicide; an overview of battered person’s syndrome and battered child syndrome in Canadian and American contexts

**DOI:** 10.3389/fpsyt.2025.1562114

**Published:** 2025-02-26

**Authors:** Oreen Mendonca, Mitesh Patel

**Affiliations:** ^1^ Faculty of Medicine, University of Toronto, Toronto, ON, Canada; ^2^ Youthdale Treatment Services, University of Toronto, Toronto Metropolitan University, Toronto, ON, Canada

**Keywords:** battered person’s syndrome, battered woman’s syndrome, battered child syndrome, abuse, homicide, self-defense, American court, Canadian court

## Abstract

Battered Person’s Syndrome (BPS) is a set of psychological symptoms experienced by victims who are victims of intimate partner violence. BPS may inform a defense in homicide cases wherein battered individuals killed their abusers. Similarly, Battered Child Syndrome (BCS) can be used as evidence to support a claim of self-defence wherein the child is the aggressor, and a care provider is the victim. Forensic psychiatrists provide expert opinion evidence regarding such claims of self-defence. A psycholegal opinion, often provided by forensic psychiatrists, can serve to identify factors that influence culpability and understanding of one’s actions at the material time of the offenses. Both BPS and BCS can be considered in the context of such assessments, however, further description and comparison of these syndromes is lacking in the current literature. The purpose of this article is to provide a succinct examination of the psycholegal parameters related to BPS and BCS in the Canadian and American contexts and to provide a perspective on how both can be compared. We also highlight several landmark cases in both Canada and the United States and provide a brief overview of the imperative role that forensic psychiatrists play in the development of such cases.

## Introduction

The etiology of violent behaviors directed from victim to aggressor has long been a matter of legal discourse. The extent to which such violent behaviors can be defined as self-defence is a question posed to forensic psychiatrists to obtain expert opinion. Such forensic opinions must be informed by extensive assessments, with a particular focus upon how violent behaviors can be informed by a history of abuse and/or trauma.

Battered Person’s Syndrome (BPS) is defined as a set of psychological symptoms that can be experienced by victims of intimate partner violence (L. E. 1). BPS has its origins as a form of self-defence and has been recognized as such by courts and forensic psychiatrists from the early 1900s (2). Battered Woman’s Syndrome (BWS) was originally utilized as a defense in homicide cases in which abused or “battered” women had killed their abuser(s).

Battered Child Syndrome (BCS) has recently been recognized as a syndrome or constellation of symptoms that inform self-defence behaviors in which the child is the aggressor, and a care provider is the victim.

Forensic psychiatrists conduct psychiatric assessments in order to provide diagnostic clarification regarding the state of mind at the time of alleged offenses. A psycholegal opinion can serve to identify factors that influence culpability and understanding of one’s actions at the material time of the offenses. Both BPS and BCS can be considered in the context of such assessments, however, further description and comparison of these syndromes is lacking in the current literature.

The purpose of this article is to provide a succinct examination of the psycholegal parameters related to BPS and BCS in the Canadian and American contexts and to provide a perspective on how both can be compared. In order to do so, the paper is divided into four key sections: in part one of the paper, we review the definitions of BPS and BCS; in part two, we explore how self-defence is defined by Canadian an American courts and its essential role in contextualizing the claim of BPS and BCS in the courts. In part three, we highlight several landmark cases in both Canada and the United States and offer a brief comparison of both syndromes. Lastly in part four, we culminate the paper by highlighting the imperative role that forensic psychiatrists play in providing psycholegal opinions related to such cases.

## Discussion

### Section 1: defining battered person’s syndrome and battered child syndrome

#### Battered person’s syndrome

BPS was first described as BWS in 1979 by psychologist Dr. Lenore Walker to educate judiciary professionals and court process of the impact of abuse, both physical and psychological, upon a woman’s perception of danger in cases of homicide (3). Though BWS refers inherently to the victim as being a woman, it is possible to extend these criteria to any victim in the context of a romantic relationship (4). For simplicity, we will refer to BPS to include all genders as potential victims of domestic abuse.

Dr. Walker describes three-phases in the cycle of violence: 1. an initial tension building phase between the victim and their abuser; 2. an acute incident in which the victim is a victim of battery, and; 3. a honeymoon phase in which the abuser seeks forgiveness for their actions (3). The honeymoon phase of the cycle often leads to the victim believing that their abuser continues to care for them and hence, deserves another chance (3). This in turn prevents them from leaving the abusive relationship. BPS has been defined as involving at least two cycles of violence (2).

BPS is often correlated with post-traumatic stress disorder (PTSD) (1, 5). PTSD is a mental health diagnosis that develops in some people who experience a dangerous event, such as a threat of death, serious injury, or sexual violence (6). Associated symptoms of PTSD include recurrent intrusive memories of the traumatic event, dissociative reactions, prolonged psychological distress, physiological reactions to cues resembling the traumatic event, persistent avoidance of stimuli associated with the event, negative cognition and mood disruption (6). Criteria for PTSD are defined in the Diagnostic Statistics Manual Version 5 (DSM-V), which is utilized primarily in both American and Canadian psychiatric contexts. In summary, PTSD is a condition that may be diagnosed when an individual experiences significant trauma, by way of direct exposure, witnessing the trauma, learning that it happened to a close relative or friend, or indirect exposure. Incidents can include exposure to death, a significant threat to death or serious injury, or actual or threatened sexual violence. Symptoms of intrusion, avoidance, altered mood, and altered reactivity are present for more than month, and the associated disturbance must cause clinically significant distress to one’s functioning (DSM-V).

BPS is defined by the presence of specific PTSD symptoms; namely, re-experiencing of traumatic events, numbing of responsiveness and hyperarousal. In addition, three symptoms that have been identified as unique to BWS include disrupted interpersonal relationships, difficulties with body image and/or somatic concerns, and sexual and intimacy problems (2). These traits are, however, not present in all victims (3). Furthermore, BPS shares the common components of learned helplessness, effects of trauma, and self-destructive coping mechanisms (3). Neither BPS or BWS is identified as a diagnosis in the DSM-V but BPS is identified in the International Classification of Diseases Nineth Revision (ICD9) (but is excluded in the 10^th^ and most recent revision).

Learned helplessness is a prominent component of BPS (3). Repeated traumatic experiences that are not contingent upon a victim’s actions and their abuser’s acts of violence lead to a sense of helplessness and the perception that they are unable to escape their circumstance. This informs the manner in which a victim is unable to perceive opportunities to escape an abusive relationship in the same manner that a non-battered victim may (7 as seen in 2, 3).

#### Battered child syndrome

BCS is defined as a cluster of symptoms that drive self-defence behaviors in abused children, culminating in an act of parricide (the killing of a parent) and/or care provider.

Literature suggests that there are three types of children who commit parricide: those that are severely abused, severely mentally ill and dangerously antisocial (8). Battered children are the most common type of adolescent parricide offender.

The concept of learned helplessness, previously discussed in BPS, also applies to BCS. The child develops the perception that their actions are unrelated to the perpetrator’s abuse and acts of violence; they establish a sense of learned helplessness in which they believe there is no way to change their situation or to escape except by killing the care-provider (8).

Battered children who engage in parricide, as compared to those children who commit homicide of strangers, often demonstrate limited social relationships, better impulse control and fewer externalizing or aggressive behaviours, as well as more instances of chronic abuse by a parent and exposure to domestic violence (8). Impulse control often develops along with hypervigilance as a coping mechanism to detect imminent signs of danger and potentially avoid further advancement of the cycle of abuse.

### Section 2: battered person’s syndrome and battered child syndrome as self-defence in the Canadian and American courts

The manner in which courts define self-defence can determine whether BCS or BPS is incorporated into cases of homicide. Although Canadian and American legal systems have developed distinct practices, both originate in the British common law and share strong similarities (9). It is common practice for forensic psychiatrists from both countries to share expertise that inform legal practices. Only Canada and America were included in this study due to their shared legal frameworks and close geographical and cultural proximity.

As such, we explore how self-defence is defined by Canadian and American courts. Following this, we summarize landmark cases of BPS and BCS in both countries and subsequently draw parallels between BPS and BCS.

#### Self-defence in Canadian versus American courts

In Canadian law, as per section 34 of Criminal Code of Canada, an act committed in self-defence is not considered a criminal offense if: (1) the person believes that a force or threat of force is being used against themselves or another person; (2) it was conducted with the purpose of defending one’s self from threat or force and; (3) the act committed is reasonable in the circumstances (10).

In 2013, this definition was updated to clarify that in order to determine if an act was reasonable, the court must consider relevant circumstances of the persons involved, and the act involved including, but not limited to, the following factors: (11):

- the nature of the force or threat;
- the extent to which the use of force was imminent and whether there were other means available to respond to the potential use of force;
- the person’s role in the incident:
- whether any party to the incident used or threatened to use a weapon;
- the size, age, gender and physical capabilities of the parties to the incident;
- the nature, duration and history of any relationship between the parties to the incident, including any prior use or threat of force and the nature of that force or threat;
  1. any history of interaction or communication between the parties to the incident;
- the nature and proportionality of the person’s response to the use or threat of force; and
- whether the act committed was in response to a use or threat of force that the person knew was lawful.

In American law, self-defence is defined at a state level and thus fluctuates across the country, though the common basis of the self-defence definition across the country has similar common elements to the traditional Canadian legal definition: (1) the person must have acted with a reasonable belief that they were in imminent danger; (2) the use of force was needed to avoid the danger, and; (3) the amount of force used is reasonable in relation to the harm threatened (12, as seen in 13).

In at least 11 states (Connecticut, Delaware, Hawaii, Maine, Maryland, Massachusetts, Minnesota, Nebraska, New Jersey, New York, Rhode Island) there is a legal duty to retreat from a situation of harm if the victim has a reasonable opportunity to do so; in the face of harm an individual must not use self-defence should they be able to escape the situation. In other states (including Pennsylvania, South Carolina, South Dakota, Tennessee, Texas, Utah, Florida), there is a “stand your ground” and “castle” statutory law, wherein an individual does not have the duty to retreat in the face of harm (14). When assessing a plea for self-defence in cases of homicide, all three of the following criteria must be met (13): (1) the victim must be in imminent danger; (2) the victim must have required the use of force to avoid danger; and (3) the victim must have used a reasonable amount of force in relation to the harm.

If a person is not in imminent danger at the time of the violent incident, both Canadian and American laws indicate that the incident does not reflect a state of self-defence.

### Section 3: Landmark cases

#### Landmark cases: battered person’s syndrome

##### USA: Michigan v. Francine Hughes Wilson

In 1977, Francine Hughes was charged with murder for setting her sleeping husband’s bed on fire. This was one of the first cases in which BPS was utilized as a defense in the United States. Francine Hughes was violently and repeatedly abused for over 13 years. She made multiple attempts to escape her plight, and sought help from lawyers, agencies and the police, however, these were unsuccessful. Following a trial by jury and expert testimony, Hughes was found not guilty and was found to be temporarily insane at the time of committing the murder (15).

##### Canada: R. v. Lavallee

In Canada, Angelique Lyn Lavallee was the first woman acquitted using a defence of BPS, in the 1990 Supreme Court of Canada case R. v. Lavallee. Lavallee shot her common law partner in the back of the head. Lavallee had a long history of abuse and was described as being a battered woman in a volatile relationship (16 as stated in 2). A psychiatric assessment revealed her ongoing state of terror and inability to escape the cycle of violence. She held the belief that killing her abuser was the only way to save her life at the material time (16). The court upheld the notion that self-defence in the traditional sense could be applied to this context of domestic abuse and that the expert evidence provided context for Lavallee’s perception of imminent danger given her history of many years of intimate partner violence and abuse.

#### Landmark cases: battered child syndrome

##### USA: state of Washington v Andrew G. Janes

One of the first cases of BCS in the American courts was that of sixteen-year-old Andrew Janes who was charged with first degree murder of his stepfather. This case was presented to the Supreme Court in 1993. Janes was determined to have experienced severe abuse and PTSD at the hands of his stepfather. Fourteen witnesses provided evidence of extensive abuse and maltreatment. A child and adolescent psychiatrist presented testimony pertaining to PTSD and the effects of prolonged abuse on a child. The psychiatrist provided an opinion that Janes had brought an end to the abuse he endured by committing homicide (17). This was the first court to acknowledge the validity of BCS as equal to that of BPS (13). Similar to BPS cases, this trial allowed expert testimony during the trial which allowed the jury to conceptualize how the victim’s history of abuse might have impacted his actions. Expert evidence and testimony additionally highlighted how victims of long-term abuse may perceive threats differently than those who do not experience such circumstances.

##### Canada: Earl Joey Wiebe case

The use of BCS as a legal defense in a Canadian context is limited, in comparison to USA. This is perhaps due, in part, to Canada’s smaller population with is approximately one tenth of the US. Of recent, BCS was successfully used in the defence of a 19-year-old Manitoban adolescent Earl who killed his stepmother in 2000 (18). Earl was found not found criminally responsible for the death of his 40-year-old step mother after choking her and lighting her bed on fire, due to mental disorder. His diagnoses included PTSD and borderline personality disorder, which was supported by the expert testimony of three mental health professionals during his trial (19).

#### A comparison of battered person’s syndrome and battered child syndrome

In BWS, an argument can be made that battered women perceive danger differently than others (3). Battered victims may not leave an abusive relationship and often perceive that homicide is the only way ensure their safety. Due to the cycle of abuse, and differences in physical strength, it is common for battered women to kill their abuser in a situation where they may not be in imminent danger (3).

Similarly, a child may not be in danger at the time of parricide, but due to past experiences of trauma and resultant hypervigilance, a child may come to predict when the next cycle of abuse is due to occur. They thus develop a belief that they are always in imminent danger and parricide may not occur when the child is in imminent danger (20, as seen in 8).

Children are more vulnerable than adults. Developmental trajectories are often impacted by abuse resulting in disrupted attachment styles. This too can influence the manner in which the child perceives threats and stressors by others. A child’s cognitive and decision-making abilities may be underdeveloped, particularly in those who have faced trauma and abuse. This can limit their ability to seek help or formulate alternatives to address their challenges, aside from violence.

It is more difficult for a child to escape an abusive environment compared to adults, not only due to their increased reliance on the aggressor to have their basic needs met, but also because there are fewer safe alternatives for children to escape to. Furthermore, children can experience stronger and numerous attachments to other individuals in the home, inclusive of a sibling or parent, which can limit the child’s willingness to leave the home (13).

In cases of both battered adults and children, there are often notable differences in physical strength between the battered individual and the aggressor. As such, it is not uncommon for victims to resort to extreme measures of violence, such as the use of a gun (2). Given the history of learned helplessness, the act of violence directed from the battered individual to the aggressor may occur when the aggressor is in a nonaggressive position, such as watching television (8). Though it can be argued that the amount of force used is thus unreasonable in the context of self-defense, considerations of the impacts of chronic abuse must thus be taken into consideration.

### Section 4: involvement by the forensic psychiatrist

Legal counsel may request a forensic psychiatric assessment to assist in determining whether an individual meets criteria for PTSD or other psychiatric diagnoses and whether symptoms and signs of BPS or BCS may be present. They may also request opinions regarding culpability and the capacity for an accused to engage in courtroom proceedings. Such assessments should be based on a meticulous review of available information and direct assessment of the victim and any other relevant parties. Both American and Canadian courts weight heavily upon expert opinion and testimony in decision-making processes.

#### Involvement of forensic psychiatrist in battered person’s syndrome

The role of expert testimony in cases of BPS is foundational to the defense. When providing such evidence it is important to establish whether a cycle of violence existed. This can include providing specific details regarding the abuse, inclusive of the type, frequency and intensity of these incidence as experienced by the victim.

As noted above, the diagnostic criteria associated with PTSD apply to BPS. Forensic assessments should utilize standardized reporting scales to determine whether diagnostic criteria for PTSD are met. It is also important to include evidence as to whether other comorbid psychiatric conditions are present. Assessments should comment on the possibility of learned helpless and biopsychosocial factors that may have caused the victim to remain in an abusive relationship (2). Furthermore, commenting on the imminence and impact that battery may have on an individual may also be important to include, as risk of an imminent attack may not be necessary for the successful use of a self-defence argument (21).

Forensic psychiatrists may provide evidence that significantly impacts outcomes related to these cases. As in the case of R. v. Lavallee, as mentioned above, the court commented that expert evidence was admissible for four purposes: (1) to challenge the myths and stereotypes of the public’s perception of battered women, (2) to detail how the woman perceived danger, (3) to explain why an abused woman may not leave an abusive relationship, and (4) to explain why the woman came to the conclusion that killing was the only way to save her own life (2). Keeping such principles in mind can assist the psychiatrist in assessing and gathering appropriate information.

#### Involvement of forensic psychiatrist in battered child syndrome

Murder of an abusive parent by a child has garnered special recognition in American courts. Four state Supreme Courts have considered expert testimony in regards to BCS; the admissibility of such testimony was based on state specific standards. For example, in two cases in Wyoming (Jahnke v. State) and Indiana (Whipple v. State), courts did not allow expert testimony because it was deemed that the testimony did not meet the requirements for admissibility. Other cases states like those in Washington (State v. Janes) and Ohio (State v. Nemeth) did consider expert testimony of BCS in their cases as admissible and relevant to actions of the victims of abuse. (22, 23).

In the 1993 BCS Ohio state Supreme court case of State v. Janes, as mentioned above, the court accepted that BWS and BCS are to be accepted as similar psychological disorders for the purposes of expert testimony (13). It followed that children are more vulnerable to the effects of abuse in comparison to adults, and as such the courts should be further compelled to accept the perspective that parricide may be a child’s only way to escape their abusive circumstance (24 as seen in 13).

The assessment of a child must consider the history and impact of abuse and/or neglect. Resilience of the child must also be considered as well as the impacts of intergenerational abuse. Psychiatric assessments should include a thorough evaluation of child welfare records, where available, as well as other collateral sources of information to determine the extent and timeline of abuse history. This can include school, pediatric and counseling records.

Attachment disorders may also be considered in BCS. Typically, children who face longstanding histories of abuse do not form secure attachments to their care-providers and display symptoms or behaviors consistent with an attachment disorder (5th ed.; DSM–5; 25). Such behaviours may include excessive familiarity with strangers or a general lack of selectivity in attachment figures (26). There may also be disorders of conduct and other challenges associated with social relatedness in various contexts. The DSM-V defines two forms of attachment disorder: reactive attachment disorder (RAD) and disinhibited social engagement disorder (DSED). The forensic psychiatrist must be adept at recognizing and diagnosing these conditions in the consideration of BCS.

## Conclusion

Battered Person’s Syndrome (BPS) and Battered Child Syndrome (BCS) serve as important considerations in cases of self-defence involving those who are battered victims turned aggressor. Given that forensic psychiatrists from both Canada and America share expertise that inform legal practice, this article provides a succinct examination of the psycholegal parameters related to BPS and BCS in the Canadian and American contexts and a unique perspective on how both can be compared. By reviewing the definitions of self-defense along with landmark cases in both countries, it is evident that forensic psychiatrists must thoroughly explore themes of learned helplessness, impacts of chronic abuse, and underlying psychiatric or attachment disorders in both syndromes. Additional consideration of the increased vulnerability of children, including mental developmental trajectory, decreased strength, and reliance for basic needs, must also be accounted for in the assessment of self-defence in BCS cases. The gravity of the forensic psychiatrist’s expert opinion and testimony in the decision-making processes of the courts and jury cannot be understated.

## Data Availability

The original contributions presented in the study are included in the article/supplementary material, further inquiries can be directed to the corresponding author/s.

## References

[1] Walker LE. Battered woman syndrome. Psychiatr Times. (2009) 26.

[2] Glancy G Heintzman M Wheeler A. Battered woman syndrome: updating the expert checklist. Int J Risk Recovery. (2019) 2:4–17. doi: 10.15173/ijrr.v2i2.3820

[3] Ferraro KJ. The words change, but the melody lingers. In: Violence Against Women. Thousand Oaks, California, USA: SAGE Publications, vol. 9. (2003). p. 110–29. doi: 10.1177/1077801202238432

[4] Hamel J. Gender-Inclusive Treatment of Intimate Partner Abuse, Second Edition: Evidence-Based Approaches. Springer (2013). p. 38–40

[5] Walker LEA. Battered woman syndrome: Empirical findings. Ann New York Acad Sci. (2006) 1087:142–57. doi: 10.1196/annals.1385.023 17189503

[6] Substance Abuse and Mental Health Services Administration (SAMHSA). Trauma-informed care in behavioral health services. In: Treatment Improvement Protocol (TIP) Series 57. U.S. Department of Health and Human Services (2014). Available online at: https://store.samhsa.gov/sites/default/files/d7/priv/sma14-4816.pdf. 24901203

[7] Walker LE. The battered woman. New York: Harper & Row (1980).

[8] Hart JL Helms JL. Factors of parricide: Allowance of the use of battered child syndrome as a defense. Aggression Violent Behav. (2003) 8:671–83. doi: 10.1016/S1359-1789(02)00103-9

[9] Davis R Prpa T. Exactly the same, but totally different: A few small but notable differences between Canada and U.S. Legal systems. Fogler Rubinoff. (2021). Available online at: https://www.foglers.com/insights/exactly-the-same-but-totally-different-a-few-small-but-notable-differences-between-canada-and-u-s-legal-systems/.

[10] Criminal Code RSC. (1985). c. C-46, s 34(1). (1985).

[11] Criminal Code RSC. (1985). c. C-46, s 34(2). (1985).

[12] LaFave Scott. . supra note 7, § 5.7(a).

[13] Ryan CS. Battered children who kill: developing an appropriate legal response. Notre Dame J Law Ethics Public Policy. (1996) 10:301–39.

[14] Self-Defense and ‘Stand Your Ground.’. National Conference of State Legislatures (2023). Available online at: https://www.ncsl.org/civil-and-criminal-justice/self-defense-and-stand-your-ground (Accessed August 27, 223).

[15] Sorrentino R Musselman M. Battered woman syndrome: is it enough for a not guilty by reason of insanity plea? Psychiatr Times. (2019) 36:7.

[16] R. v. Lavallee. (1990). Available online at: https://www.canlii.org/en/ca/scc/doc/1990/1990canlii95/1990canlii95.html. 1990canlii95. CanLII, 95(SCC) (Accessed June 05, 2023).

[17] State v. Janes. (1993).

[18] Battered child syndrome led to killing of stepmom - judge. CBC News [Internet]. (2001). CBC News. Available from: https://www.cbc.ca/news/canada/battered-child-syndrome-led-to-killing-of-stepmom-judge-1.268416 (Accessed July 16, 2023).

[19] Foss K. Abuse led to slaying, judge rules. The Globe and Mail (2001). Available online at: https://www.theglobeandmail.com/news/national/abuse-led-to-slaying-judge-rules/article4157432/ (Accessed June 15, 2023).

[20] Goodwin MR. States are beginning to recognize that abused children who kill their parents should be afforded the right to assert a claim of self-defense. Southwestern Univ Law Review. (1996) 25:429–60.

[21] Tang K. Battered woman syndrome testimony in Canada: its development and lingering issues. Int J Offender Ther Comp Criminol. (2003) 47:618–29. doi: 10.1177/0306624X03257519 14661383

[22] Skier Law Firm. Is “Battered Child Syndrome” A Defense to Murder? Montgomery, Alabama, USA: Skier Law Firm. (2019). Available online at: https://www.skierlawfirm.com/blogs/5900/is-battered-child-syndrome-a-defense-to-murder/.

[23] Szymanski LA. Battered child syndrome as defense for murder of abusive parent. National Center for Juvenile Justice. (2000) 5. Available online at: https://www.ncjfcj.org/publications/battered-child-syndrome-as-defense-for-murder-of-abusive-parent/.

[24] Janes, 822. P.2d at 1241.

[25] American Psychiatric Association. Diagnostic and statistical manual of mental disorders, 5th. Washington, DC: American Psychiatric Association Publishing (2013). doi: 10.1176/appi.books.9780890425596.

[26] Hanson RF Spratt EG. Reactive attachment disorder: What we know about the disorder and implications for treatment. Child Maltreatment. (2000) 5:137–45. doi: 10.1177/1077559500005002005 11232086

